# Care access and utilization among medicare beneficiaries living with Parkinson’s disease

**DOI:** 10.1038/s41531-023-00523-y

**Published:** 2023-07-10

**Authors:** Caroline Pearson, Alex Hartzman, Dianne Munevar, Megan Feeney, Rachel Dolhun, Veronica Todaro, Sheera Rosenfeld, Allison Willis, James C. Beck

**Affiliations:** 1 NORC at the University of Chicago, Illinois, 60603 USA; 2 Parkinson’s Foundation, New York, NY 10022 USA; 3 Michael J. Fox Foundation, New York, NY 10022 USA; 4 University of Pennsylvania Perelman School of Medicine, Philadelphia, PA 19104 USA; 5 Present Address: Peterson Center on Healthcare, New York, NY 10022 USA; 6 Present Address: California State University, Stanislaus, Turlock, CA 95382 USA; 7 Present Address: VOZ Advisors, New York, NY 10001 USA

**Keywords:** Health care, Neurological disorders

## Abstract

An estimated 90% of people living with Parkinson’s disease (PD) in the US are covered by Medicare health insurance. How these beneficiaries use and engage the health care system is important to understand in the face of a rapidly growing PD population. Here, we analyzed health care utilization patterns of those with a PD diagnosis enrolled in Medicare in 2019. By our estimates, PD beneficiaries number 685,116 or 1.2% of the total Medicare population. Compared to the overall Medicare population, 56.3% are male (vs 45.6%), 77.9% over age 70 (vs 57.1%), 14.7% people of color (vs 20.7%), and 16.0% are rural residents (vs 17.5%). Our analysis identified significant disparities in care. Surprisingly, 40% of PD beneficiaries (*n* = 274,046) did not see a neurologist at all during the calendar year and only 9.1% visited a movement disorder specialist (MDS). Few Medicare beneficiaries diagnosed with PD use recommended services such as physical, occupational, or speech therapy. People of color and rural residents were least likely to access a neurologist or therapy services. Despite 52.9% of beneficiaries being diagnosed with depression, only 1.8% had a clinical psychology visit. Our findings emphasize the need for further research on population-specific barriers to accessing PD-related health care.

## Introduction

Within the United States, an estimated 89% of those diagnosed with PD are eligible for government-provided Medicare health insurance either because of their age (65 years of age and older) or prolonged disability status^1^. However, few studies have examined the health care utilization patterns of people living with PD on Medicare and how demographic differences, especially for groups that have been historically underrepresented in research, impact utilization of health care services^2,3^.

Inconsistent symptom presentation and disease progression, as well as lack of biomarker or objective clinical diagnostic test to diagnose disease, creates a challenge for diagnosing and treating PD, especially for physicians with less expertise in movement disorders^4^. As such, diagnosis and care are ideally managed by general neurologists and/or movement disorder specialists in an outpatient setting^5,6^. Previous research finds that movement disorder specialists more accurately diagnose PD, especially in earlier stages and atypical presentations, than neurologists without subspecialty training; benefits of early detection can include reduced risk of disease progression and improved quality of life^7^. Additionally, PD specialist involvement in the management of PD patient care has been found to improve the patient experience in all care settings and stages of care^6^. Given the impact of both motor and non-motor symptoms of PD, treatment for PD should include pharmaceutical interventions, along with rehabilitative therapy, and mental health services^8^.

As part of a broader portfolio of research on how people living with PD access health care and information, this analysis uses Medicare program data to explore the demographic characteristics, including gender, age, race and ethnicity, and rural residency (“urbanicity”), and PD-related health care utilization. These analyses use comprehensive data on Medicare beneficiaries and services made available by the Centers for Medicare & Medicaid Services. The implications of this study are particularly important when considered along with the significant rates of growth of incident Parkinson’s disease in the US population, which increased more than 50% over the past decade (2012–2020), as well as the increase in the American population and associated projected growth of the Medicare population, which is expected to grow by 20.3% between 2021 and 2029^9–11^.

## Results

### Prevalence and demographics of Parkinson’s disease in the medicare population

In 2019, there were 64,430,729 beneficiaries enrolled in the US Medicare system (Table 1). We restricted our analysis to those beneficiaries who had at least one claim with an ICD-10 diagnostic code of G20 indicating Parkinson’s disease (808,107 beneficiaries, comparable to prior Medicare-specific projections and age proxied U.S. and international projections^1,12–14^.) Subsequently, we limited analyses further to beneficiaries who were continuously enrolled in full coverage Medicare Fee-for-Service (FFS) (Parts A and B) or a Medicare Advantage (Part C) plan during the 2019 calendar year and had observable physician information included in their claims or encounter records. This identified 685,116 or 1.2% of the corresponding total Medicare population with at least one ICD-10 code diagnosis of “Parkinson’s disease”.

**Table 1:** Exclusion criteria for study population *2019 Medicare Parkinson’s disease population* 2019.

	Excluded *n*	Remaining *n*
Beneficiaries enrolled in 2019	–	64,430,729
Beneficiaries with at least 1 G20 (“Parkinson’s disease”) diagnosis in 2019	63,622,622	808,107
Beneficiary deaths in 2019	80,423	727,684
Beneficiaries enrolled for only part of 2019	8698	718,986
Beneficiaries with partial FFS/MA coverage in 2019	19,870	699,116
Beneficiaries missing provider information in all Medicare claims in 2019	14,000	685,116
*2019 Medicare beneficiaries with Parkinson’s disease* *(study population)*	**685,116**	

This represents the final number of beneficaries included in the analysis after exclusions listed above.

People living with PD enrolled in Medicare tended to be older relative to the Medicare population as a whole (77.9% versus 57.1% over age 70, respectively). Medicare beneficiaries with PD were also more likely to be male (56.3%) than the broader Medicare population, which is 45.6% male and 54.4% female. The PD population was only slightly less likely to reside in rural areas (16.0%) compared to other Medicare beneficiaries (17.5%). Rurality was defined using beneficiary county of residence code linked to USDA Rural-Urban Continuum Codes (RUCC). 39.5% of beneficiaries living with PD were enrolled in Medicare Advantage plans compared to 37.3% of Medicare beneficiaries overall.

People living with PD were 2.3% Asian, 5.9% Black, 2.6% Hispanic, 0.3% North American Native, and 85.3% White (Table 2). People of color tend to be underrepresented among beneficiaries living with PD relative to the overall Medicare population (14.7% vs 20.7%). This is particularly notable with Black PD beneficiaries who represent 5.9% of the population vs. 10.5% in the overall Medicare population. Whether this is due to underdiagnosis, delay in diagnosis, reduced survival^15^, or truly lower prevalence of PD among these populations is not known.

**Table 2:** Prevalence of Parkinson’s disease diagnosis by demographic characteristics in Medicare 2019.

	Medicare beneficiaries with Parkinson’s disease (2019)		Total Medicare beneficiaries* (2019)	
	*n*	%	*n*	%
Total population	685,116	–	56,888,373	–
Sex				
Female	299,549	43.7	30,967,181	54.4
Male	385,567	56.3	25,921,184	45.6
Age				
70 and under	151,411	22.1	24,404,014	42.9
Over 70	533,705	77.9	32,484,359	57.1
Race and ethnicity				
Asian	15,947	2.3	1,450,377	2.5
Black	40,097	5.9	5,952,719	10.5
Hispanic	17,911	2.6	1,723,507	3
North American Native	2,268	0.3	258,074	0.5
Other	13,891	2	1,170,014	2.1
Unknown	10,776	1.6	1,185,755	2.1
White	584,226	85.3	45,147,927	79.3
Urbanicity				
Rural	109,451	16.0	9,951,576	17.5
Non-rural	575,665	84.0	46,930,412	82.5
Medicare enrollment				
Fee-for-Service (FFS)	414,401	60.5	35,651,994	62.7
Medicare Advantage (MA)	270,715	39.5	21,236,379	37.3
Co-Occurring Chronic Disease				
Depressed and/or Anxious	362,314	52.9	15,553,742	27.3
Neither Depressed nor Anxious	322,802	47.1	41,334,631	72.7

### Health care utilization–physician services

The majority (60.0%) of Medicare beneficiaries with PD had at least one visit with a neurology specialist (i.e., general neurologist or a movement disorder specialist (MDS)) in 2019. Specifically, 50.9% of the population had at least one visit with a general neurologist (MDS = 0, GN ≥ 1, Table 3), but did not see an MDS. Only 9.1% of individuals with PD visited an MDS at least once in 2019. However, 40% (274,046 people living with PD) instead sought care from a primary care physician or did not see a physician at all for their PD during the year (Table 3).

**Table 3:** Utilization of physician services by Medicare beneficiaries with PD by demographic characteristics 2019 (percent).

	Movement Disorders Neurologist (MDS)	General Neurologist	Primary Care Provider (PCP)	No Neurology Specialist nor PCP
	MDS >0	MDS = 0, General Neurology > 0	MDS = 0, General Neurology = 0, PCP > 0	MDS = 0, General Neurology = 0, PCP = 0
Total population	9.1	50.9	29.2	10.8
Sex				
Female	8.4	48.9	31.3	11.5
Male	9.8	52.4	27.5	10.3
Age				
70 and under	11.3	51.0	24.7	13.0
Over 70	8.5	50.8	30.4	10.2
Race and Ethnicity				
Asian	7.8	49.1	33.5	9.7
Black	5.4	43.9	36.7	14.0
Hispanic	4.6	48.6	36.8	10.0
North American Native	5.0	44.5	36.4	14.2
White	9.4	51.3	28.5	10.7
Other	10.3	54.6	26.4	8.8
Unknown	15.4	56.8	19.1	8.7
Urbanicity				
Rural	7.2	47.7	32.9	12.2
Non-Rural	9.5	51.5	28.4	10.6

Almost all differences across demographic groups reported are significantly different to *p* < 0.01. Not statistically different: General Neurologist between age groups.

There were significant demographic differences in physician utilization. As shown in Table 3, female beneficiaries living with PD sought care from MDS and general neurologists at lower rates than male beneficiaries. 8.4% of female beneficiaries living with PD visited an MDS at least once in 2019 compared with 9.8% of male beneficiaries. 48.9% of female beneficiaries that did not have an MDS visit in 2019 had at least one general neurology visit compared to 52.4% of male beneficiaries (Table 3). Medicare beneficiaries under the age of 70 were more likely to have had at least one MDS visit in 2019 (11.3%) than older beneficiaries (8.5%). Reported differences are significant to *p* < 0.01; please see tables for further detail.

Asian, Black, Hispanic, and North American Native beneficiaries with PD utilized specialty care at significantly lower rates than White beneficiaries. 7.8% of Asian beneficiaries, 5.4% of Black beneficiaries, 4.6% of Hispanic beneficiaries, and 5.0% of North American Native beneficiaries had at least one MDS visit in 2019 compared to 9.4% of White beneficiaries. 51.3% of White beneficiaries did not use MDS care but had at least one general neurology visit compared with 49.1% of Asian beneficiaries 43.9% of Black beneficiaries, 48.6% of Hispanic beneficiaries, and 44.5% North American Native beneficiaries (Table 3).

Individuals residing in rural areas were less likely to receive care from an MDS than their urban counterparts. Only 7.2% of rural residents saw an MDS at least once in 2019, compared to 9.5% of urban residents. Rural residents were also less likely than non-rural residents to visit a general neurologist if they did not visit an MDS (47.7% of rural residents vs. 51.5% of non-rural residents) (Table 3).

### Health care utilization–therapy and mental health services

Since non-pharmacological therapies for treating symptoms of PD are crucial to manage the disease, we examined the number of PD Medicare beneficiaries that used physical, occupational, and speech-language therapy providers (Table 4) where PD was listed on the claim. In 2019, 20.3% of the population of Medicare beneficiaries living with PD used physical therapy, 9.5% used occupational therapy, and 7.5% used speech-language therapy. Additionally, despite 52.9% of Medicare beneficiaries with PD having a diagnosis of depression and/or anxiety, only 1.8% of these individuals had at least one clinical psychology visit and 3.9% had at least one psychiatry visit.

**Table 4:** Percent utilization of therapy services by Medicare beneficiaries with PD by demographic characteristics 2019.

	Physical therapy	Occupational therapy	Speech-language therapy
Total population	20.3	9.5	7.5
Sex			
Female	19.8	10.2	6.9
Male	20.6	8.9	7.9
Age			
70 and under	17.3	7.3	6.2
Over 70	21.1	10.1	7.8
Race and Ethnicity			
Asian	14.9	5.8	5.1
Black	16.9	9.7	6.7
Hispanic	12.3	5.4	4.2
North American Native	18.9	7.9	6.7
White	20.9	9.8	7.7
Other	19.4	6.9	6.3
Unknown	22.0	8.1	7.5
Urbanicity			
Rural	19.3	9.6	7.4
Non-Rural	20.5	9.5	7.5

Almost all differences across demographic groups reported are significantly different to p < 0.01. Not statistically different: Occupational therapy between Black and White, Physical therapy and Speech-Language therapy between North American Native and White, Speech-Language therapy between ‘unknown’ race/ethnicity and White, and Occupational therapy and Speech-Language therapy between rural and non-rural urbanicity.

As seen with specialist care, there were significant demographic differences in utilization of physical, occupational, and speech-language therapy among Medicare beneficiaries living with PD (Table 4). Male beneficiaries with PD were more likely to use physical therapy (20.6%) or speech-language therapy (7.9%) than female beneficiaries (19.8% and 6.9%, respectively). Individuals over age 70 were more likely to use therapy services than those under the age of 70. Asian, Hispanic, and North American Native individuals used all therapy services at rates below average. 9.7% of Black beneficiaries used occupational therapy, a higher rate than the 9.5% average. Rural and non-rural Medicare beneficiaries living with PD accessed therapy services at similar rates (Table 4).

Medicare beneficiaries with PD who received specialist care during the year were generally more likely to use therapy and mental health services, with those being treated by an MDS being the most likely to access these services (Table 5, Supplementary Table 2). Medicare beneficiaries who had at least one MDS visit in 2019 were most likely to use therapy and mental health service providers in the same year—13.1% used occupational therapy, 33.2% used physical therapy, 13.1% used speech-language therapy (Table 5); 3.8% used clinical psychology and 3.7% used psychiatry (Supplementary Table 2). Individuals who did not see an MDS but used general neurology services at least once per year were more likely to used physical and speech-language therapy and clinical psychology services than those who did not seek care from neurology specialists (i.e., either an MDS or general neurologist) (Table 5 and Supplementary Table 2).

**Table 5:** Percent utilization of therapy services as a function of physician utilization 2019.

	Occupational therapy	Physical therapy	Speech-language therapy
	%	%	%
**MDS** *MDS > 0*	13.1	33.2	13.1
**General Neurology** *MDS = 0, General Neurology >0*	9.2	23.2	7.7
**PCP** *MDS = 0, General Neurology = 0, PCP>0*	11.4	16.3	7.4
**None** *MDS = 0, General Neurology = 0, PCP = 0*	2.6	6.4	1.7

All differences across primary provider utilization are significantly different to *p* < 0.01.

This represents the final number of beneficaries included in the analysis after exclusions listed above.

## Discussion

This study uses large-scale administrative data to measure Parkinson’s disease prevalence from the most inclusive, population-based U.S. health care database to identify the size, demographic characteristics, and health care utilization patterns of the population of Medicare beneficiaries living with PD. An important strength of this study is the substantial size of the Medicare dataset, including the totality of the Fee-for-Service and Medicare Advantage populations included in a single study. The use of this dataset allows for a robust analysis of the demographic characterization of the Medicare beneficiary population living with PD, and more reliable estimates of health care utilization than previous studies that rely on data collected from hospitals and physician groups or limited solely to Medicare FFS. Additionally, past research has largely focused on the prevalence of PD in White people, males, and those living in non-rural areas^2,3,16^. In comparison, this study analyzed a population of over 685,000 Medicare beneficiaries, reducing bias related to underrepresentation that is more prevalent in survey research and in location-specific utilization research^3^.

We also find that male beneficiaries were 1.22 times more likely to have PD than female beneficiaries. Several studies find that PD is more common in men than women, by male-to-female ratios ranging between 1.1 and 2.7 in populations 50 years of age and older^17^. A previous study of the Medicare population found PD was 1.39 times more prevalent in male than female beneficiaries^15^.

Our study finds that nearly 250,000 people living with PD (40%)—a startling number—do not access the care of a specialist for their PD, a particularly complex disease to medically manage^18^. PD care by clinicians with neurology training (general neurologists and MDS neurologists) has been shown to yield best outcomes and survival^19^. Nearly 50% of PD beneficiaries access the care of a neurologist. In addition, only 9.1% of people living with PD (approximately 62,300) access the care of an MDS neurologist, recognized for providing care with the highest outcomes for those with PD^7^. Part of the reason for such a significant number of individuals not accessing specialist care could be that neurologists are more likely found in population dense urban areas^20^; however, so too are PD patients. A more likely reason is that there is an alarming shortage of neurologists to provide the specialist care needed for those with PD^21,22^. Compounding this is the relative dearth of MDS neurologists; there are about 660 MDS in the US^23^, which equates to an average of one MDS per 1038 Medicare beneficiaries living with PD. Like neurologists, MDS clinicians are not uniformly distributed and are often found in urban areas^23^. To overcome the lack of specialist clinicians, there has been not only a recognition of the need for more neurologists^21^, but also the recommendation for incorporating advanced practice practitioners into the clinic^24,25^. In that vein, the Parkinson’s Foundation is initiating specialty training for advanced practice practitioners with the goal of increasing access to those with specialized PD clinical training^26^.

When examining access to and utilization of specialist physician services between rural and urban areas, we find significantly lower utilization for those living in rural areas. 7.2% of rural residing Medicare beneficiaries utilized MDS care, compared to 9.5% of non-rural residents and 9.1% of all beneficiaries with PD. Utilization of general neurology care among those who did not utilize MDS care is also lower for rural residents (47.7%) than non-rural residents (51.5%). This may be explained by access barriers related to distance from specialists who are not uniformly distributed across the US^20^. Studies suggest these distance barriers reduces utilization of PD specialty care, particularly for lower-income populations and those living in rural areas^3,6^. For example, access to MDS is especially challenging since only 6 out of 660 MDS practice in rural areas^23^. Despite disparities in accessing specialist care, individuals with PD living in rural areas received therapy services at similar rates to people in non-rural areas, which may suggest specialist shortages in rural areas, but adequate referrals to therapy providers from primary care providers.

Individuals under the age of 70 were more likely to utilize specialty care and less likely to use therapy services. This may be due to the prevalence of newer diagnoses in the population under 70 compared to those over 70, which is confirmed in various studies on population-level PD onset^12,14,17^. Individuals earlier in their disease progression are more likely to be in the diagnostic phase of their disease which requires multiple specialist evaluations and may include testing various supplemental care offerings to find the combination of care that best manages their symptoms^5,7^.

We find that 52.9% of Medicare beneficiaries with PD have a diagnosis of depression and/or anxiety, but only 1.8% of these individuals had at least one clinical psychology visit and 3.9% had at least one psychiatry visit. This may be because there is a gap in mental health coverage in current Medicare policies which creates cost and network barriers for beneficiaries attempting to access needed mental health services^27^. Due to the lack of Medicare insurance coverage of mental health services, beneficiaries may seek and pay for mental or behavioral health services out-of-pocket, which is thereby not captured in the administrative claims data used for this analysis.

Although the use of therapy services such as physical, occupational therapy, and speech-language therapy and mental health services are considered key interventions for the management of PD, utilization of these services remains low among Medicare beneficiaries living with PD. Our study finds that utilization of specialist physician care is a predictor of utilization of therapy and mental health services. There were significant differences in utilization of therapy and mental health providers depending on the type of physician from which an individual sought care. Medicare beneficiaries who visited an MDS in 2019 were most likely to also have at least one physical therapy visit (33.2%), occupational therapy visit (13.1%), and/or speech-language therapy visit (13.1%). These differences in utilization could be the result of provider practice patterns, the disease severity of the MDS patient population, or because some MDS providers have collocated physical therapy and occupational therapy services. Utilization of mental health services for Medicare beneficiaries who get PD care from an MDS shows a similar pattern—3.8% had a clinical psychology visit, 3.7% had at least one psychiatry visit. It is likely that this is because MDS are specifically trained to coordinate care for their patients with PD, and studies have found that integration of an MDS in all stages and settings of care is associated with an improvement in patient experience and quality of life^4–7^.

Our analysis reveals significant gaps in how many Medicare beneficiaries with PD access health care, compared to recommended best practice. It further reveals persistent health disparities for women, people of color, and rural residents—each of whom may face challenges with PD diagnosis and access to treatment. Finally, the analysis demonstrates differences in referral patterns among physician types, with patients seeing an MDS being more likely to use therapy and mental health services to treat the common symptoms of PD, indicating these clinicians may more aptly serve patients with PD given the importance of maintaining mobility and mental wellbeing for this patient population. This suggests opportunities to improve access to specialized care by expanding PD-specific training for general neurologists, advanced practice practitioners, and pursuing strategies to improve access to care across demographic groups and geographies. PCPs might be supported by encouraging referrals to MDS and general neurologists at the time of a suspected PD diagnosis.

## Methods

This retrospective observational study assesses the prevalence of Parkinson’s disease in the 2019 Medicare population, and healthcare utilization of Medicare beneficiaries living with Parkinson’s disease.

### Data

This study was conducted using 2019 Medicare enrollment data, Fee-for-Service claims (Parts A and B), and Medicare Advantage (Part C) encounter data obtained from the Centers for Medicare & Medicaid Services (CMS). Demographic and location data was retrieved from Medicare Beneficiary Summary Files. Diagnostic, provider specialty, and utilization information was derived from administrative claims and encounter data. Some Part C encounter data lacked information on provider identity or specialty, where possible this was imputed from data available in Part A and B claims. Beneficiaries enrolled in Part C whose encounter data were entirely lacking provider information were excluded from demographics and utilization measures. The NORC Institutional Review Board granted exemption of informed consent for research activities conducted under this protocol as the study is not considered human subjects research under the Common Rule.

### Prevalence

Prevalence of Parkinson’s disease in the Medicare population during the study year, 2019, is defined as the number of Medicare beneficiaries continuously enrolled in the same calendar year with one or more Medicare claims with a primary or secondary diagnosis of PD. Parkinson’s disease was identified using International Classifications of Diseases, 10th Revision, Clinical Modification (ICD-10-CM) code G20. Medicare beneficiaries with partial Medicare FFS (Parts A and B) or Medicare Advantage (Part C) coverage, and beneficiaries with missing provider information in all medical claims were not included in the study. ICD-10 coding for Parkinson’s disease is more specific than ICD-9 codes used in prior studies and does not include secondary Parkinsonism diagnoses^12^. Of those identified with PD, 83.8% of beneficiaries had more than one medical service claim with a PD diagnosis in 2019 and 72.1% had more than two claims; demographics differ based on the number of applicable diagnostic codes with Black, Hispanic, and rural residents being more likely to have exactly one G20 code in the year. (Supplementary Table 1). We opted for the broadest possible inclusion criteria of at least one G20 code to include racial and ethnic groups and rural residents that may experience greater barriers to accessing PD care, but this definition may include some individuals who are undergoing testing but do not have an actual PD diagnosis in our results.

Of note, our identified Medicare prevalence of 685,116 (after exclusions), about 1.2% of the total population, is consistent to the approximately 450,000 observed in the 2000–2005 Medicare FFS dataset by Willis et al.^15^. (which was 1.6% of the FFS population at the time). Also like Willis et al.^15^, we observe that Asian, Black, and Hispanic people tend to be less well represented among PD patients than the overall Medicare population. It is not known if this is indicative of disparities in screening and diagnosis of PD among these racial and ethnic groups, or if it points to a potential genetic or environmental component of PD that more often affects White people. Previous research confirms that PD is most commonly found in White people, but also finds that racial and ethnic differences in incident PD cannot be explained by differences in age, sex, income, insurance, or healthcare utilization. Therefore, research suggests that the prevalence of PD may be explained by biological differences, environmental factors, or other social determinants^15,28^. Research also suggests that Black and Hispanic people are less likely to use health care services or self-report symptoms than White people, which may also contribute to underdiagnosis in these populations^2,29,30^.

### Healthcare services utilization

Utilization was calculated for physician services (neurology—movement disorder specialist, general neurology, and primary care), therapy services (physical, occupational, and speech-language therapy), and mental health services (clinical psychology and psychiatry). Healthcare utilization was summarized as a function of age, sex, race and ethnicity, and rurality derived from data found in the Master Beneficiary Summary File. Chronic condition flags for anxiety and depression indicators were applied for FFS enrollees; these flags were reconstructed using diagnostic information on encounter data for MA enrollees.

Additionally, therapy utilization and mental health service utilization was analyzed as a function of physician service utilization.

### Statistical analyses

Demographic characteristics and prevalence of Parkinson’s disease in the Medicare population were summarized with descriptive statistics. Inferential statistics were used to describe and quantify cohort differences in healthcare services utilization. Outcomes were compared statistically between cohorts using chi-squared tests. Differences between cohorts were considered statistically significant at *p* < 0.01. Excel software was used for statistical analysis.

### Limitations

This study has several limitations. Estimates of PD prevalence for the total Medicare population may be low given that measurements of the population are based on a strict criterion for a one-year period (2019) in which individuals must have been continuously enrolled, had full Medicare coverage, and sought care from a provider that recorded their PD diagnosis on at least one billed outpatient or physician office service claim. As a result, individuals who did not seek care from a Medicare provider in 2019 are not captured in these estimates; relatedly, this study also did not measure length of PD diagnoses, an important consideration to address in subsequent research. Additionally, our analysis relies on medical claims billed for Parkinson’s disease and other services and is subject to limitations of the accuracy and use of those codes. Therefore, prevalence could be underestimated in cases where diagnoses were incompletely or inaccurately captured in claims, or among individuals who had interruptions or changes to their Medicare enrollment or coverage. Likewise, the categorization of race may not truly reflect the PD population due to the limitation that Medicare beneficiaries are only able to supply a single response to the question of racial/ethnic categorization.

Another limitation is the potential for both under- and over-estimation of the PD services utilization among the population in this study. First, this study likely underestimates utilization of health care services in the population of Medicare beneficiaries living with PD, due to missing provider data, inaccurate and/or incomplete coding, and interruptions or changes to Medicare enrollment and/or coverage. However, a source of potential overestimation is that we assume that individuals living with PD are seeking care from MDS and general neurologists primarily for the management of their PD as a PD diagnosis (ICD-10-CM code G20) must be included on the claim for us to measure it. That said, this study does not explicitly account for co-existing conditions or services rendered during physician visits. This overestimation effect is likely greater when considering therapy utilization given that physical and occupational therapy, and speech-language therapy are commonly used to manage other conditions that are common among older adults, though all utilization measured in this study had to include a PD diagnosis on the claim.

Finally, the decision to focus on data from 2019 stemmed from a desire to concentrate on health care consumption patterns in a single calendar year prior to the short-term disruption in care seeking and care access patterns brought on by the COVID-19 pandemic in 2020. The year 2019 was chosen because it was the most current year of available Medicare Advantage data at the time the research was conducted. Future studies should examine additional years of data with a focus on time since PD diagnosis, along with medication claims and usage, to ascertain more detail about the PD population in Medicare. Nevertheless, given these limitations, these analyses reveal important findings about the utilization of healthcare services by the PD population within Medicare and provides a baseline to guide more nuanced estimates of PD prevalence as they become available.

### Reporting summary

Further information on research design is available in the Nature Research Reporting Summary linked to this article.

## Supplementary information

Supplemental Tables

Reporting Summary

## Data Availability

Data supporting this study are not publicly available. Due to CMS policy, the authors are unable to provide the data used in this study. Data can be requested through ResDAC’s CMS Data Request Center.
