# Supplementary material for: Care access and utilization among medicare beneficiaries living with Parkinson’s disease
Source: NPJ Parkinsons Dis. 2023 Jul 10;9:108. doi: 10.1038/s41531-023-00523-y (PMC10333279; doi:10.1038/s41531-023-00523-y)
Supplement: Supplementary file 1 — Supplemental Tables [file 41531_2023_523_MOESM1_ESM.pdf]

Supplemental Tables for Care Access and Utilization Among Medicare Beneficiaries Living with Parkinson's Disease

Supplemental Table 1. Demographics of Medicare Enrollees by Number of Claims with a PD (G20) ICD-10 Diagnostic Code, 2019

	At Least 1 G20 Dx	At Least 2 G20 Dx	Exactly 1 G20 Dx
	%	%	%
Sex			
Female	43.8	43.4	47.1
Male	56.2	56.6	52.9
Age			
70 and under	22.1	21.6	26.1
Over 70	77.9	78.4	73.9
Race and Ethnicity			
Asian	2.3	2.3	2.4
Black	5.9	5.7	7.7
Hispanic	2.7	2.6	2.9
North American Native	0.3	0.3	0.4
Other	2.0	2.0	1.8
Unknown	1.6	1.6	1.4
White	85.2	85.4	83.4
Urbanicity			
Rural	15.9	15.7	17.7
Non-Rural	84.1	82.2	82.3

Supplemental Table 2. Utilization of Mental Health Services as a Function of Physician Utilization, 2019

	Clinical Psychology	Psychiatry
	%	%
MDS<br>*MDS >0*	3.8	3.7
General Neurology<br>*MDS=0, General Neurology >0*	1.5	2.3
PCP<br>*MDS=0, General Neurology=0, PCP>0*	0.6	2.2
None<br>*MDS=0, General Neurology=0, PCP=0*	0.7	3.14
